# Pulmonary Alveolar Microlithiasis: A Unique Case of Familial PAM Complicated by Transplant Rejection

**DOI:** 10.1155/2021/6674173

**Published:** 2021-04-05

**Authors:** Austin Helmink, Samir Atiya, Ernesto Martinez Duarte

**Affiliations:** Department of Pathology and Microbiology, University of Nebraska Medical Center, Omaha, NE 68198, USA

## Abstract

**Background:**

Pulmonary alveolar microlithiasis (PAM) is a rare lung disease characterized by the deposition of calcium phosphate microliths or calcospherites, within the alveolar airspace. Typical imaging findings demonstrate a “sandstorm” appearance due to bilateral, interstitial sand-like micronodularities with basal predominance.

**Methods and Results:**

We describe an unusual case of a 48-year-old male with severe, familial PAM ultimately treated with a bilateral lung transplant.

**Conclusions:**

PAM is a rare lung disease caused by a mutation in the *SLC34A2* gene, which encodes for a sodium-phosphate cotransporter in type II alveolar cells, leading to accumulation of intra-alveolar phosphate causing microlith formation. PAM has an indolent course but can progress to chronic hypoxic respiratory failure, ultimately requiring lung transplant, the only known effective treatment.

## 1. Introduction

Pulmonary alveolar microlithiasis (PAM) is a rare lung disease characterized by the deposition of calcium phosphate microliths or calcospherites, within the alveolar airspace [1]. It was first described approximately 150 years ago as numerous laminated bodies in the alveolar spaces and interstitium [2]. PAM is secondary to mutations of the “solute carrier family 34 member 2” (*SLC34A2*) gene, which encodes a sodium-phosphate cotransporter, NPT2b, found on type II alveolar cells [2–4]. There is a blockade in phosphate reabsorption in PAM, leading to excess alveolar phosphate that binds with calcium to form calcium phosphate crystals [5]. Of the more than 1000 cases reported worldwide [6], many are discovered incidentally on chest imaging, and when symptomatic, the clinical course is generally slowly progressive [1, 7, 8]. Typical imaging findings demonstrate a “sandstorm” appearance due to bilateral, interstitial sand-like micronodularities with basal predominance [7, 9]. Here, we report the case of a 48-year-old male with severe, familial PAM ultimately treated with a bilateral lung transplant.

## 2. Case Report

A 48-year-old Hispanic male presented to our institution after being diagnosed with PAM six years prior via surgical lung biopsy during a video-assisted thoracoscopic surgery (VATS) procedure. He became oxygen-dependent a year after. After three years, the patient underwent a lung transplant evaluation but did not receive one due to a history of alcohol abuse, lack of social support, and poor esophageal motility. More recently, the patient was hospitalized multiple times for respiratory failure requiring high flow oxygen and steroids. At presentation, the patient was on 10 L oxygen and still experiencing dyspnea at rest. He also endorsed periodic sharp chest pain with exertion, peripheral edema, and fatigue. Medical history was notable for pulmonary artery hypertension treated with sildenafil, ambrisentan, and Lasix. He also had a history of insulin-dependent diabetes, chronic pancreatitis, gastroesophageal reflux disease (GERD), hypertension, and alcohol abuse. Family history was notable for two sisters, out of a total of eight siblings, who have also been diagnosed with PAM with a presumed genetic origin, though so far, the patient refuses to get tested, and further information is unavailable. These siblings have been controlled with medical treatment and had not had lung transplants.

**Figure 1:**
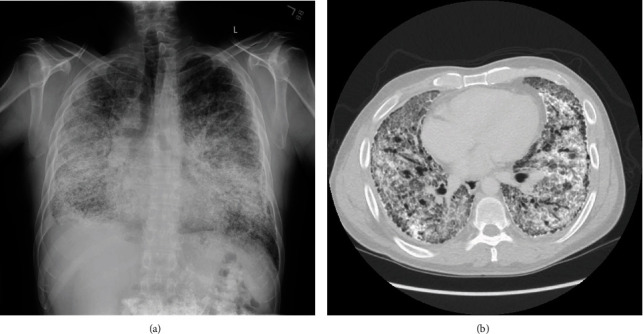
Radiologic findings consistent with PAM. (a) Chest X-ray demonstrating diffuse bilateral interstitial hyperdensities with bibasilar predominance consistent with severe interstitial lung disease. (b) Transverse view of chest CT showing diffuse ground-glass opacities, peripheral cystic change, and diffuse areas of fine calcification.

Following the presentation to our institution, the patient was evaluated and referred to the lung transplant program. Pulmonary function testing demonstrated a normal FEV1/FVC ratio with decreased FEV1, FVC, and TLC. The patient was unable to remove his oxygen supply long enough to perform DLCO. Chest X-ray demonstrated the classic “sandstorm” appearance of severe bilateral interstitial hyperdensities with a basilar predominance (Figure 1(a)). A chest CT scan showed chronic interstitial lung disease with diffuse ground-glass opacities, peripheral cystic changes, and numerous areas of calcification (Figure 1(b)).

**Figure 2:**
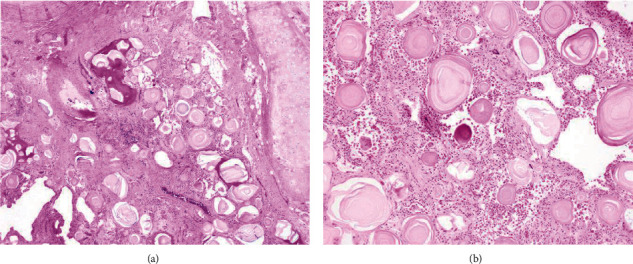
Histologic examination of explanted lung tissue. (a) 4x H&E sections of bilateral lung parenchyma demonstrated similar findings consisting of numerous microliths filling alveolar spaces, diffuse interstitial fibrosis, and focal osseous metaplasia. (b) 10x higher magnification showing the characteristic concentric calcification of the microliths and focal osseous metaplasia.

The patient underwent a bilateral lung transplant. A successfully treated pneumonia complicated his immediate postoperative course, and he went home three weeks after surgery. Gross examination of the explanted lungs demonstrated anthracotic pleural surfaces with diffuse fibrous adhesions. Upon sectioning, the parenchyma showed diffuse fibrosis and bilateral diffuse calcifications. Histologic analysis revealed numerous bilateral intra-alveolar laminated eosinophilic bodies in association with marked interstitial fibrosis. Multiple foci of osseous metaplasia and chronic inflammation with germinal center formation were identified (Figures 2(a) and 2(b)). These changes were more prominent at the bases, although involvement was diffuse and bilateral.

Multiple readmissions complicated the patient's posthospitalization course. Nine days after his initial discharge, he presented with fever and cough. Workup showed pleural effusions and evidence of sternal dehiscence. Bronchoscopy showed anastomotic necrosis, and biopsy showed evidence of minimal acute rejection (grade 1A). The patient underwent sternal hardware replacement. One week following discharge, he presented with hypoxemia during pulmonary rehabilitation therapy and was readmitted. A chest CT was suspicious for pneumonia. Repeat biopsies showed patchy perivascular chronic inflammation with no acute rejection (grade 0). However, C4d immunostaining showed strong linear staining of several small interstitial vessels, and the patient tested positive for donor-specific antibodies with increasing mean fluorescence intensity (MFI) for HLA-DQ7. The patient was given IVIG and underwent five rounds of plasmapheresis. Repeat biopsies following plasmapheresis were negative for evidence of rejection. FEV1 and FVC have both improved from pretransplant levels. DQ7 MFI has decreased significantly, but the patient remains on maintenance intravenous immunoglobulin (IVIG).

Due to PAM's family history and known familial nature of this disease, genetic testing was recommended but has not been completed at the time of this publication.

## 3. Discussion

PAM is a rare, autosomal recessive interstitial lung disease characterized by the widespread deposition of calcium phosphate crystals (hydroxyapatite microliths) within alveolar airspaces, also known as microliths, calcipherites, or calcospherites [7]. These patients have no known calcium metabolic dysfunction but rather a mutation of the “solute carrier family 34 member 2” (SLC34A2) gene, which encodes a sodium-phosphate cotransporter, NPT2b, found on type II alveolar cells [1–4]. Transporter dysfunction leads to excessive phosphate accumulation within the airspace and subsequent microlith formation [1]. This mutation is familial in about one-third of cases with autosomal recessive inheritance [7]. In familial cases, inheritance is horizontal, characteristic of autosomal recessive diseases. Castellana et al. report that when three or more siblings are affected in familial cases, they are usually females, and a vertical inheritance pattern is associated with evidence of consanguinity [1, 6]. Currently, 27 different mutations of the *SLC34A2* gene encoding the transporter have been identified [1]. PAM has a worldwide distribution, but it is more prevalent in Asia and Europe, with the most cases being in Turkey, followed by China, Japan, India, Italy, and the United States [6]. Most affected patients are diagnosed in the second through fourth decades with no apparent gender predisposition [1, 10]. PAM's clinical presentation is highly variable, with most patients being asymptomatic in the early stages of the disease with an insidious onset and slow progression over decades [1, 10]. As microliths accumulate, pulmonary fibrosis develops, leading to impaired gas exchange and hypoxia, and pulmonary hypertension [1, 11]. Symptoms may progress to include dyspnea on exertion and eventually at rest and dry cough, chest pain, and cyanosis in severe disease, eventually leading to respiratory failure [12, 13]. The extrapulmonary disease has not been well characterized but affects the testes, kidney, gallbladder, sympathetic nerve chain, and heart [6, 11, 14, 15]. Diagnosis is typically by chest radiographic imaging, including X-ray and CT. Imaging studies frequently reveal fine, sand-like micronodular interstitial hyperdensities with basilar predominance [1]. A four-tier grading system for radiologic severity has recently been proposed [16]. The degree of clinicopathologic dissociation is often significant in PAM, with extensive radiologic progression and minimal associated clinical symptoms [16]. It is essential to differentiate PAM from other diseases with similar radiographic appearances, including miliary tuberculosis, fungal infection, pneumonia, sarcoidosis, amyloidosis, pulmonary alveolar proteinosis, pulmonary hemosiderosis, metastatic calcification, and pneumoconiosis [7, 16]. Pulmonary function testing can aid in the diagnosis, though PFTs are often normal early in the disease. In advanced disease, testing typically shows a restrictive pattern with reduced DLCO [16]. Routine blood tests have no value in PAM diagnosis and generally are normal [1, 16]. PAM's definitive diagnosis lies on typical radiographic findings plus genetic testing or, in the absence of testing, radiography with tissue diagnosis [16]. Bronchoalveolar lavage can help recover microliths but is otherwise unremarkable and not used for diagnosis [12]. Lung tissue is available via either a transbronchial approach with forceps or cryobiopsy or surgical biopsy, either open or video-assisted [1, 12]. Histologic features typical of PAM are intra-alveolar, spherical, lamellated, calcium-phosphate crystals, usually <1.0 mm but can be up to 5.0 mm as those from our case [16]. Additionally, these microliths may undergo osseous metaplasia [17]. The interstitium may show fibrosis, inflammation, and calcification [17]. These characteristic findings generally strike on H&E, but trichrome stain highlights the microliths [16].

Empiric treatment of PAM has been unsuccessful [1]. Steroids are ineffective, and the use of bisphosphonate disodium etidronate has shown mixed results [10, 18, 19]. The only effective treatment at this time is a lung transplant, though clear guidelines of the timing of the transplant are not clear due to disease rarity [1, 20]. Both single and double lung transplantation have been effective, with no documented recurrence cases in transplanted lungs [1, 21].

## 4. Conclusion

Here, we present an interesting case of severe, end-stage familial PAM ultimately treated with a double lung transplant. PAM is a rare lung disease caused by a mutation in the *SLC34A2* gene, which encodes a sodium-phosphate cotransporter in type II alveolar cells. The mutated transporter leads to the accumulation of intra-alveolar phosphate, causing calcium-phosphate microlith formation. Microliths impair gas exchange and induce further pathologic changes, including interstitial fibrosis and pulmonary hypertension. PAM has an indolent course but can progress to chronic hypoxic respiratory failure, ultimately requiring lung transplant, the only effective treatment.
